# DNA Fragment Length Analysis Using Machine Learning Assisted Vibrational Spectroscopy

**DOI:** 10.1021/acsmeasuresciau.6c00099

**Published:** 2026-08-10

**Authors:** Rashad Fatayer, Waseem Ahmed, Irene Szeto, Stephen-John Sammut, Ganapathy Senthil Murugan

**Affiliations:** † Optoelectronics Research Centre, Building 53, 7423 University of Southampton, University Road, Southampton, Hampshire SO17 1BJ, United Kingdom; ‡ Perioperative and Critical Care Theme, NIHR Southampton Biomedical Research Centre, Southampton Centre for Biomedical Research, University Hospital Southampton NHS Foundation Trust, Tremona Road, Southampton, Hampshire SO16 6YD, United Kingdom; § The Breast Cancer Now Toby Robins Research Centre, 378499 The Institute of Cancer Research, Mary-Jean Mitchell Green Building, Chester Beatty Laboratories, 237 Fulham Road, London, SW3 6JB, United Kingdom; ∥ The Royal Marsden NHS Foundation Trust, Fulham Road, London, SW3 6JJ, United Kingdom

**Keywords:** vibrational spectroscopy, ATR-FTIR spectroscopy, Raman spectroscopy, DNA fragment length, DNA, machine learning, deep learning

## Abstract

DNA length analysis is essential for genomic workflows including next-generation sequencing and fragmentomics-based diagnostics. Conventional approaches typically require large, expensive instrumentation and sample-destructive protocols with long processing times. Here we present a rapid, label-free approach integrating vibrational spectroscopy with deep learning to quantify DNA fragment length distributions. We demonstrate that ATR-FTIR and Raman spectroscopy capture length-dependent spectral features arising from phosphate backbone, nucleobase, and structural vibrations. Machine learning models trained on spectra acquired from purified monodisperse DNA (50–300 bp) predicted DNA length with high accuracy (*R* ^2^ = 0.92–0.94), with multimodal fusion improving performance to *R* ^2^ = 0.96. A convolutional neural network trained on DNA mixtures comprising molecules of different lengths also successfully recovered their fragment length profile. Transfer learning enabled adaptation to biological samples, achieving low prediction error (RMSE = 0.18–7.2%, Δμ = 12 bp). Importantly, the method requires only 4 μL of sample and 15 min of passive drying, with no consumables beyond cleaning materials, and allows full sample recovery. These results demonstrate the initial feasibility of vibrational spectroscopy as a rapid, label-free, and nondestructive approach for estimating DNA fragment length distributions.

## Graphical abstract

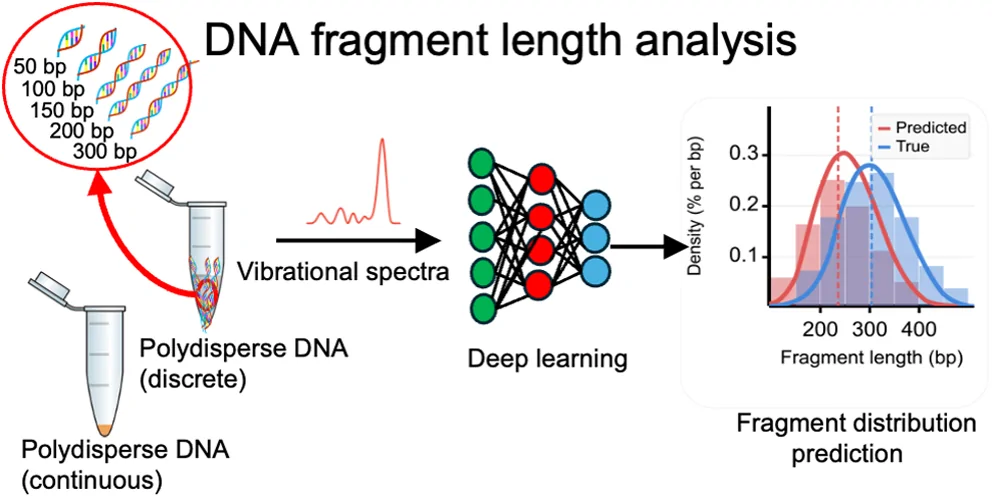

## Introduction

The quantification of DNA fragment length is a critical step in many genomic workflows, including next generation sequencing library preparation.^1,2^ Beyond sequencing, DNA fragment length distribution in biofluids such as blood is emerging as a key biomarker in several diagnostic contexts.^3^ For example, in cancer, DNA released by tumor cells into the circulation is shorter (90–150 base pairs) than that released by normal cells (∼167 base pairs),^4^ and this difference can be detected by sequencing. Similarly, DNA specific length profiles allow for the differentiation of fetal DNA from maternal DNA in prenatal screening,^5^ and the identification of viral DNA in infectious disease monitoring.^6^

Conventional methods for fragment length analysis include gel electrophoresis, which estimates length based on DNA migration through a gel matrix,^7^ but offers limited resolution and requires lengthy, multistep workflows. Higher resolution technologies such as automated capillary electrophoresis and sequencing offer higher accuracy and precision but are more expensive, require larger instrumentation and involve more stringent sample preparation protocols.^8–10^ These constraints limit the accessibility of high-resolution DNA fragment length quantification, particularly in laboratories operating under budgetary limitations. To address this, we explored an alternative approach that bridges the gap between accessible, low-resolution methods and high-cost, high-precision platforms. We designed and evaluated a novel methodology based on vibrational spectroscopy, specifically Attenuated Total Reflectance-Fourier Transform Infrared (ATR-FTIR) and Raman spectroscopies, combined with machine learning to enable label-free, nondestructive DNA fragment length quantification. This technology probes the vibrational modes of chemical bonds, producing spectral features that reflect the underlying molecular structure.^11–13^

In this proof-of-principle study (Figure 1), we present a novel method to investigate whether length dependent structural features in DNA can be detected using vibrational spectroscopy. We show that variations in band intensities and wavenumber positions carry fragment length information that allows accurate quantification of DNA fragment lengths. Furthermore, we demonstrate that a 1D convolutional neural network (1D-CNN) can estimate fragment length proportions in mixtures from ATR-FTIR spectra with low error. Collectively, these findings suggest that machine learning assisted vibrational spectroscopy may provide a rapid and low-cost option for fragment length analysis, particularly in settings where cost-effective and fast workflows are required.

**Figure 1:**
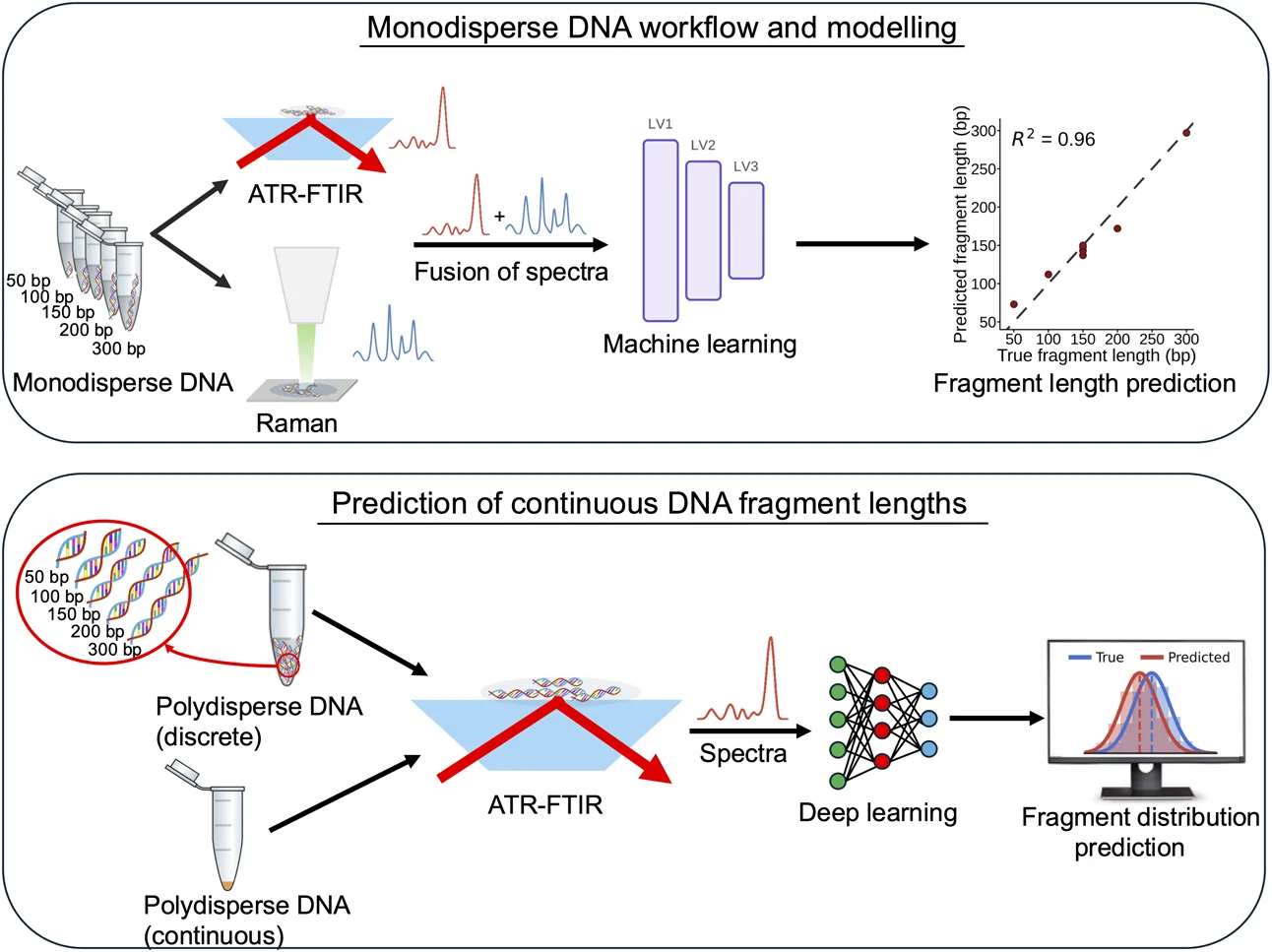
Study overview. Monodisperse DNA samples containing single fragment lengths were analyzed using ATR-FTIR and Raman spectroscopy. Spectra were combined using low-level data fusion and used as input to a Partial Least Squares Regression (PLSR) model for DNA fragment length prediction. For polydisperse DNA samples, two types of sample types were prepared. The first comprised defined mixtures of monodisperse DNA fragments combined in proportions to generate discrete polydisperse DNA mixtures. The second consisted of polydisperse DNA produced by shearing of genomic DNA, producing a continuous fragment length distribution. Discrete polydisperse samples were analyzed using ATR-FTIR spectroscopy, and the resulting spectra were used to train a one-dimensional convolutional neural network (1D-CNN). Transfer learning approach was used (from discrete polydispersed to continuous polydispersed) to quantify DNA fragment length distribution in biological samples.

## Methods and Materials

### DNA Samples

Purified DNA fragments of defined lengths 50 bp (catalogue number SM1421), 100 bp (SM1441), 150 bp (SM1601), 200 bp (SM1611), and 300 bp (SM1621); 0.5 μg/μL (in TE buffer) were purchased from ThermoFisher Scientific. For the monodisperse experiments, these samples were used in their undiluted form and were directly pipetted to the ATR crystal for spectral acquisition. To generate a comprehensive data set for 1D-CNN model development, polydisperse DNA mixtures were prepared from the purified DNA fragments using an extreme vertices experimental design. This design strategy, implemented through Minitab software, explored the five-fragment mixture space by efficiently sampling combinations that would enable accurate quantification of proportions. The resulting 34 sample design set comprised: (i) pure component samples, (ii) binary mixtures containing two fragments, (iii) ternary mixtures with three fragments, (iv) quaternary mixtures with four fragments, (v) centroid mixtures with all five fragments in equal proportions, and (vi) additional mixtures with all five fragments in varying proportions. The coverage from one component samples to multi fragment mixtures allowed comprehensive model training and evaluation across diverse fragment distribution profiles. Each mixture was prepared by the volumetric combination of fragment stock solutions at a fixed total volume of 12 μL. Since all stock solutions were obtained at identical concentrations, the relative fragment abundance in each mixture was defined by the volumetric ratios used during mixing, with exact compositions reported in .

Rat genomic DNA was prepared using the Monarch HMW DNA extraction kit for tissue (NEB #T3060) following manufacturer’s instructions. Rat mammary tissues were pestle-homogenized and lysed in HMW gDNA tissue lysis buffer and proteinase K (600 μL lysis and 20 μL proteinase K per 15 mg tissues) at 56 °C for 5 h agitated at 1400 rpm in a thermo mixer. The lysate was then digested with 10 μL of RNase A at 56 °C for 10 min agitated at 1400 rpm 300 μL of protein separation solution was added and the tube was inverted for 1 min and spun for 10 min at 16000g. The upper phase was transferred using a 1000 μL wide-pore pipet tip to a new 2 mL tube and 2 DNA capture beads and 550 μL isopropanol were added. The tube was slowly inverted for 30 times allowing 5–6 s for each inversion. The liquid was discarded and 500 μL of gDNA wash buffer was added to the beads. The tube was inverted for 3 times and the wash buffer was removed. This wash step was repeated once. The beads were poured onto a bead retainer in a collection tube. After the tube was spun briefly to remove residual wash buffer. The beads were poured from the retainer into a new 2 mL tube. 100 μL elution buffer II was added to the beads and incubated for 5 min at 56 °C agitated at 300 rpm in a thermal mixer. The beads and elution volume were poured onto the saved retainer in a 1.5 mL tube. The tubes were spun at 12000g for 30 s. The eluate was saved and resuspended up and down with a wide-pore tip for 10 times. This genomic DNA was subsequently sheared using Covaris S220 following manufacturer’s instructions. Briefly, 5 μg of DNA in 0.1 M Tris-HCl (pH 8.0) was put in a microTUBE-50 (Covaris PN520166) in a final volume of 55 μL for each condition. The water level was set at 10 at 7 °C. Target BP (Peak) was set to 150, 200, 250, 300, or 350 bp. The fragment length distribution for each condition was analyzed using the Agilent 4200 TapeStation system with HS D1000 ScreenTape.

### ATR-FTIR Spectroscopy

ATR-FTIR spectra were acquired using an Agilent Cary 670 FTIR spectrometer equipped with a liquid nitrogen cooled MCT detector and a MIRacle 9 bounce reflection ATR fitted with a diamond coated ZnSe crystal. For each measurement, 4 μL of DNA was pipetted onto the ATR crystal and allowed to air-dry at room temperature for approximately 15 min to form a uniform thin film. Spectra were collected across the 700–6000 cm^–1^ range at a spectral resolution of 4 cm^–1^, with 64 scans averaged per acquisition to enhance the signal-to-noise ratio. Each sample was measured in triplicate, with the ATR cleaned between each measurement and nine repeated scans performed per sample before proceeding to the next composition.

### Raman Spectroscopy

Raman measurements were performed using a Renishaw inVia micro-Raman spectrometer equipped with a 532 nm excitation laser and a 20× objective lens. While 785 nm excitation is generally preferred for biological Raman measurements to minimize fluorescence, the 532 nm laser was the available source; fluorescence background was further mitigated through the use of CaF_2_ substrates. For sample preparation, 2 μL of each DNA fragment length solution was deposited onto a CaF_2_ substrate and dried at room temperature

Spectra were collected over the 600–1800 cm^–1^ range. After focusing the microscope objective on the dried deposit, Raman spectra were acquired at the outer edge of the sample using 10 accumulations with a 10 s integration time, yielding 15 spectra per position. The laser position was then adjusted, and an additional 15 spectra were collected to ensure adequate coverage of the dried sample’s surface.

### Data Preprocessing

All preprocessing procedures were carried out after splitting the data into training and testing subsets to prevent data leakage. Optimized parameters derived exclusively from the training data were subsequently applied to the test set. For ATR-FTIR spectra, the analysis was truncated to 750–1800 cm^–1^, as the diamond ATR crystal introduces strong absorbance between ∼1800–2700 cm^–1^, limiting interpretability in this region. For Raman spectra, the full acquired wavenumber range was retained without truncation and cosmic ray artifacts were removed using Renishaw’s WiRE software. Vector normalization was then performed across the most informative spectral regions (FTIR*:* 750–1800 cm^–1^ and Raman*:* 600–1800 cm^–1^) to account for intensity variations. A manual three-point baseline correction was applied to all spectra to eliminate background drift, followed by a Savitzky-Golay second order derivative transformation to enhance spectral resolution and resolve overlapping features.

### Machine Learning

PLSR was used as a supervised learning approach to quantify individual DNA fragment lengths from their corresponding spectra. This method is particularly well suited for high dimensional spectroscopic data, as it effectively addresses multicollinearity by extracting latent variables that maximize the covariance between the features and the target response variable (fragment length).

To prevent data leakage, data splitting was performed in a manner that respected the structure of our measurements. In spectroscopic experiments, replicates refer to repeated measurements of the same kind of sample following physical replacement or repositioning (e.g., removing a DNA sample from the ATR crystal and reapplying it). In contrast, repeats refer to multiple spectra acquired from the same physical sample without repositioning (e.g., multiple spectra collected from the same DNA spot). Since repeats from the same replicate originate from the same underlying sample, they are not statistically independent and were therefore kept together during train/test splitting to avoid overestimating model performance.

For the FTIR data set, data splitting was performed on a replicate basis where the first two replicate sets were used for training and cross-validation and the third was held out as an independent test set. For the Raman data set, splitting was based on physical sampling location to prevent data leakage. For each sample measured, spectra acquired around one laser spot were used exclusively for model training, whereas those from the proximity of a second spot were reserved for independent testing. For both the ATR-FTIR and Raman data sets, the train/test ratio was maintained at approximately 80/20, while ensuring that nonindependent repeat spectra were not split between the training and test sets.

Model optimization and latent variable selection were performed on the training set to prevent overfitting with the optimal number of PLS components being determined using 10-fold cross validation in combination with Van der Voet’s comparison approach and F-test. This method objectively compares the cross-validation prediction errors (mean squared error) between models with *n* and *n + 1* components. The first nonsignificant comparison (*p ≥ 0.05*) was taken as the criterion for selecting the optimal number of components, ensuring a parsimonious model that captures relevant variance without fitting to noise.

### Performance Metrics

PLSR model performance (monodisperse DNA) was evaluated on individual spectra, with each spectrum treated as an independent prediction. The coefficient of determination (*R* ^2^) was computed across all individual test spectra, reflecting the proportion of variance in fragment length explained by the model. Root mean square error (RMSE) was also computed across all individual test spectra.

For the 1D-CNN model (polydisperse discrete mixtures), two complementary performance metrics are reported: (i) per-class RMSE was computed separately for each of the five fragment length classes and (ii) distribution weighted mean shift (Δμ) was computed at the level of mixture composition rather than individual spectra. For each mixture composition in the test set, spectra were averaged to yield a single mean predicted proportion vector per composition. The weighted mean fragment length of this predicted distribution was then compared to the calculated weighted mean of the true (known) proportion vector for that mixture composition. Δμ was defined as the absolute difference between the predicted and true weighted mean.

For the transfer learning model applied to biological (sheared genomic DNA) samples, predictions take the form of seven-element proportion vectors, representing the predicted percentage of DNA within each predefined fragment length bin (25–50 bp, 50–100 bp, 100–150 bp, 150–200 bp, 200–250 bp, 250–300 bp, and 300–350 bp), derived from gel electrophoresis reference measurements. Per-sample RMSE was computed using the averaged spectra from the five independent test samples. Additionally, the distribution-weighted mean fragment-length shift was also reported.

### Confidence Intervals

To quantify uncertainty in the reported test-set RMSE, a nonparametric percentile bootstrap was applied to the paired true and predicted values from the held-out test set. After model training and prediction, the observed RMSE was first computed on the full test set. The paired true and predictions were then resampled with replacement 10,000 times, with each resample having the same size as the original test set. RMSE was recalculated for each resample, producing a bootstrap distribution of RMSE values. The 95% confidence interval was defined as the 2.5th and 97.5th percentiles of this distribution. The resulting interval is reported alongside the observed RMSE as RMSE [lower bound, upper bound] in base pairs for the PLSR model and proportion for the transfer learning model.

### Convolutional Neural Network (CNN) Development

All deep learning model development, including baseline training, hyperparameter optimization, and transfer learning, was conducted on the University of Southampton’s high-performance computing (HPC) cluster, Iridis X. Training jobs were executed on NVIDIA L4 GPU nodes using the CUDA-enabled TensorFlow backend, with cuDNN providing GPU-accelerated operations. Reproducibility was ensured using deterministic TensorFlow settings, fixed random seeds, and strict group-wise data splits that kept all replicates from each sample together, preventing data leakage and ensuring test performance reflected true generalization to unseen samples.

Hyperparameter optimization was performed using the Hyperband algorithm to efficiently explore learning rates, convolutional filters, kernel sizes, dropout levels, regularization strengths, dense layer architectures, neuron counts, and data augmentation parameters. This approach allocated computational resources to the most promising configurations that minimized validation loss.

To increase model robustness, the purified DNA training set was augmented 6-fold using controlled perturbations () inspired by the spectral variation framework of Bjerrum et al. (2017).^14^ Realistic instrumentation variation was simulated through three transformation types applied in combination: (i) intensity scaling, using a factor sampled uniformly between 0.99–1.01 times the training set standard deviation, (ii) vertical offset implemented as an additive shift sampled uniformly between −0.01 and +0.01 absorbance units scaled by the spectral training set standard deviation, and (iii) uniform noise introduced as random perturbations up to 0.001 absorbance units, scaled by the training set standard deviation.

The optimized architecture for the 1D-CNN consisted of a single convolutional layer, one hidden dense layer, and a linear output layer predicting five fragment percentages. The model was trained using the Adam optimizer with Huber loss () and a 20% validation split. To enforce physically meaningful outputs, all model predictions were passed through a custom Constrained Prediction Layer that normalized outputs to sum to 100%.

### Transfer Learning for Biological Samples

A two-stage transfer learning framework was developed to adapt spectral representations learned from polydisperse (discrete) DNA mixtures to polydisperse (continuous) biological DNA samples. In Stage 1, the pretrained 1D-CNN served as a feature extractor with all convolutional layers (including weights, biases, and regularization parameters) frozen to preserve learned DNA features. A new prediction head comprising dense layers was trained on top of the frozen feature extractor, allowing the model to learn biologically relevant mappings from purified fragment features to real DNA fragment proportions without disrupting the pretrained convolutional filters.

After Stage 1 convergence, all layers were unfrozen in Stage 2 and the entire network was fine-tuned end-to-end using a reduced learning rate (0.0001 vs 0.001). This fine-tuning allowed convolutional filters to adapt to biological sample complexity while retaining the learned structural features, thereby improving generalization.

Model optimization employed a customized Wasserstein-MSE loss function combining the Wasserstein distance and mean squared error (). The Wasserstein component (Earth Mover’s Distance) considered the spatial ordering of fragment length intervals, penalizing predictions that misplaced mass across distant bins, while the MSE component penalized bin-specific errors, together encouraging accurate recovery of both interval-wise magnitudes and overall distributional shape.

### Feature Interpretation

To identify the most informative spectral regions contributing to DNA fragment length quantification, regression coefficients from the PLSR models were plotted against wavenumber. Large absolute coefficient values indicate wavenumbers whose signal varies most systematically with fragment length, providing a direct measure of each spectral variable’s predictive contribution. This approach also served to validate the chosen spectral windows (750–1800 cm^–1^ for ATR-FTIR; 600–1800 cm^–1^ for Raman), confirming that the analysis was focused on meaningful DNA vibrational bands while excluding regions dominated by noise or buffer contributions.

### DNA Fragment Length Distributions

Fragment length distributions for the biological DNA samples were generated through shearing using a Covaris S220 instrument. Instrument parameter sets (TP settings) were used to target specific modal fragment lengths, including TP150, TP200, TP250, TP300, and TP350. For example, the TP150 setting was configured to produce a dominant fragment population centered around approximately 150 bp, while progressively higher TP settings were used to shift the fragment length distributions toward larger sizes.

Gel electrophoresis was used to check the resulting fragment length distributions and served as the reference measurement. Fragmentation experiments were repeated once and produced comparable electropherograms across duplicate preparations for all TP settings, with the exception of TP350, which exhibited broader distributions and increased inter-replicate variability.

### Prediction and Visualization of Fragment Length Distributions

For the purified DNA mixtures, the 1D-CNN was trained to predict the percentage contribution of each discrete fragment length (50, 100, 150, 200, and 300 bp). For the biological data, the transfer learning model predicted the percentage of DNA assigned to predefined fragment length intervals (25–50 bp to 300–350 bp), derived from gel electrophoresis size distributions. In both cases, the raw model outputs are binned proportion vectors expressed as percentages.

To visualize the underlying fragment length distribution as a continuous curve, the discrete proportions were first converted to density units (% per bp) by dividing each proportion by its corresponding bin width. For the purified DNA mixtures, where fragments are discrete rather than interval-based, a uniform bin width was applied consistently across all fragment lengths to enable density conversion. For the biological samples, the true bin width of each interval was used directly. A Gaussian distribution was then parametrized using the weighted mean and standard deviation of the bin proportions and evaluated on a fine grid of fragment lengths to produce a smooth curve for plotting.

### Data Integration (Fusion)

For each sample, we extracted the informative window from both modalities (IR: absorbance 750–1800 cm^–1^ and Raman: counts 600–1800 cm^–1^). Each data set was independently normalized (min-max normalization within its own wavenumber range) to prevent one modality from dominating due to scale differences. We then performed true axis concatenation where the IR wavenumber axis was kept in its original wavenumber range (750–1800 cm^–1^), and the Raman axis was shifted by a constant offset so that it begins immediately after the IR band, creating a single combined axis with no overlapping. Specifically, the shift is given as concat_shift = (max IR wavenumber - min Raman shift). The fused CSV file thus contains a continuous spectral vector in which the IR section is followed immediately by the shifted Raman section. To preserve physical interpretability, we also stored the original (unshifted) wavenumber/shift in an OrigWavenumber column and tagged each row with Source ∈ {IR, Raman}.

## Results and Discussion

### DNA Fragment Length in Monodisperse Solutions

To establish proof of principle, we first investigated whether vibrational spectroscopy could discriminate between monodisperse DNA solutions comprising a single fragment length (measured in base pairs, bp). Solutions containing DNA fragments of five discrete lengths (50, 100, 150, 200, and 300 bp), each at an identical concentration, were analyzed using ATR-FTIR spectroscopy (Methods). Following spectral averaging (Figure 2a), fragment length-dependent variations in both absorbance intensity and peak position were observed across the range 750–4000 cm^–1^.

**Figure 2:**
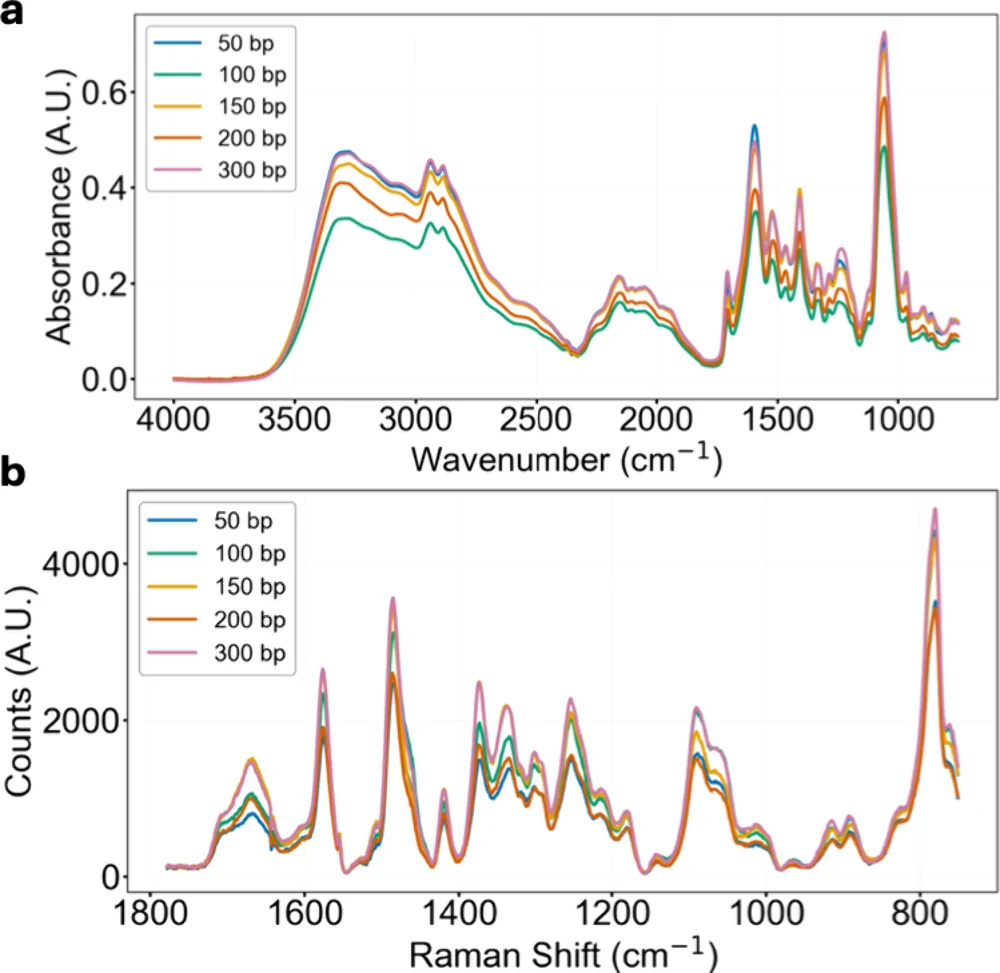
Spectroscopic analysis of monodisperse DNA solutions containing different fragment lengths. (a) Averaged FTIR and (b) averaged Raman spectra of DNA fragments (50–300 bp).

The most prominent DNA associated features were observed in (1) the symmetric PO_2_^–^stretching band (∼1080 cm^–1^), which showed the largest change in intensity across fragment lengths, (2) the asymmetric PO_2_^–^ stretching region (∼1220–1250 cm^–1^), and (3) nucleobase associated vibrations from C=O, C=N, and C=C bonds (∼1500–1700 cm^–1^).^15–18^ Within the ∼1250 cm^–1^ band, shorter fragments displayed a shift toward higher wavenumbers. This behavior is consistent with the structural organization of DNA, as these spectroscopic bands originate from sugar phosphate backbone vibrations. Fragment length-dependent differences were also observed within the nucleobase region (∼1500–1700 cm^–1^), likely reflecting base stacking interactions^19,20^ associated with increasing structure regularity in longer fragments. Variations between fragment lengths were also detected around ∼3300 cm^–1^ within the broad N–H and O–H stretching regions consistent with changes in hydrogen bonding associated with DNA hydration and base groups.^21^

We subsequently analyzed these DNA monodisperse solutions using Raman spectroscopy (Figure 2b). Raman spectroscopy was included as a complementary vibrational modality to assess whether the fragment length-dependent spectral features observed by ATR-FTIR were also detectable using an independent vibrational technique. The Raman spectra showed that the strongest band observed was at ∼785 cm^–1^, which is associated with O–P–O stretching vibrations, together with cytosine and thymine ring breathing modes.^22^ Strong contributions were also observed in the 1300–1800 cm^–1^ region typically associated with nucleobase vibrations.^23,24^ A further prominent feature occurred at ∼1085 cm^–1^, corresponding to symmetric PO_2_^–^ stretching,^25^ consistent with the ATR-FTIR findings where this band was also identified as having strong fragment length dependence. Additional bands were observed between 1250 cm^–1^ and 1375 cm^–1^ originating from cytosine, thymine, and adenine vibrations, as well as purine ring stretching modes. Other regions seen from 1485 cm^–1^ to 1580 cm^–1^ are assigned to adenine, guanine, and cytosine ring stretching modes, with a sharp band at ∼1660 cm^–1^ reflecting C=O stretching of cytosine, thymine and guanine.^23^

Across all DNA with various fragment lengths, the Raman spectral features remained consistent in overall shape but showed length dependent intensity variations, most notably in the ∼1085 cm^–1^ and ∼1450 cm^–1^ bands. Wavenumber shifts were also observed relative to the ∼1250 cm^–1^ nucleobase associated region, however this effect was not as prominent as observed in ATR-FTIR.

In summary, both ATR-FTIR and Raman spectra displayed fragment length-dependent spectral changes in monodisperse DNA solutions, with differences arising from backbone, nucleobase and hydrogen bonding-related vibrations. However, these spectral differences should be interpreted within the context of the defined DNA fragments analyzed here. Previous studies have shown that DNA base composition and sequence can influence ATR-FTIR and Raman spectra, particularly for shorter oligonucleotide sequences and within nucleobase-associated spectral regions.^26–28^ Therefore, while the changes observed here are consistent with fragment length-dependent effects under the controlled conditions used in this study, possible contributions from sequence-dependent spectral variation cannot be fully excluded.

### Multivariate Analysis of Monodisperse DNA Fragment Length Solutions

To explore whether the spectral features identified by ATR-FTIR and Raman were sufficient to predict DNA fragment length in monodisperse solutions, we first split the data into training and test sets and applied Partial Least Squares Discriminant Analysis (PLS-DA) to each modality separately. PLS-DA was used to incorporate the fragment length labels and extract latent variables (LVs) that maximize class separation based on the underlying spectral features. Five PLS latent variables were retained for the PLS-DA models. For both ATR-FTIR and Raman data sets, the second and third latent variables provided the strongest class separation.

Across both modalities, the PLS-DA latent-space projections showed separation between the five DNA fragment-length groups (Figure 3a and b). For ATR-FTIR, the clustering was more structured, with the samples following a ordered progression from 50 to 300 bp primarily along LV2. This indicates that the spectral variance captured by the model could be systematically related to fragment length. In contrast, the Raman PLS-DA projection showed less orderly clustering. While separation between fragment lengths was still observed, the group positions did not follow a clear progression with increasing fragment length and there was greater overlap between several classes. This suggests that the fragment-length-dependent variation was represented more consistently in the ATR-FTIR spectra than in the Raman spectra.

**Figure 3:**
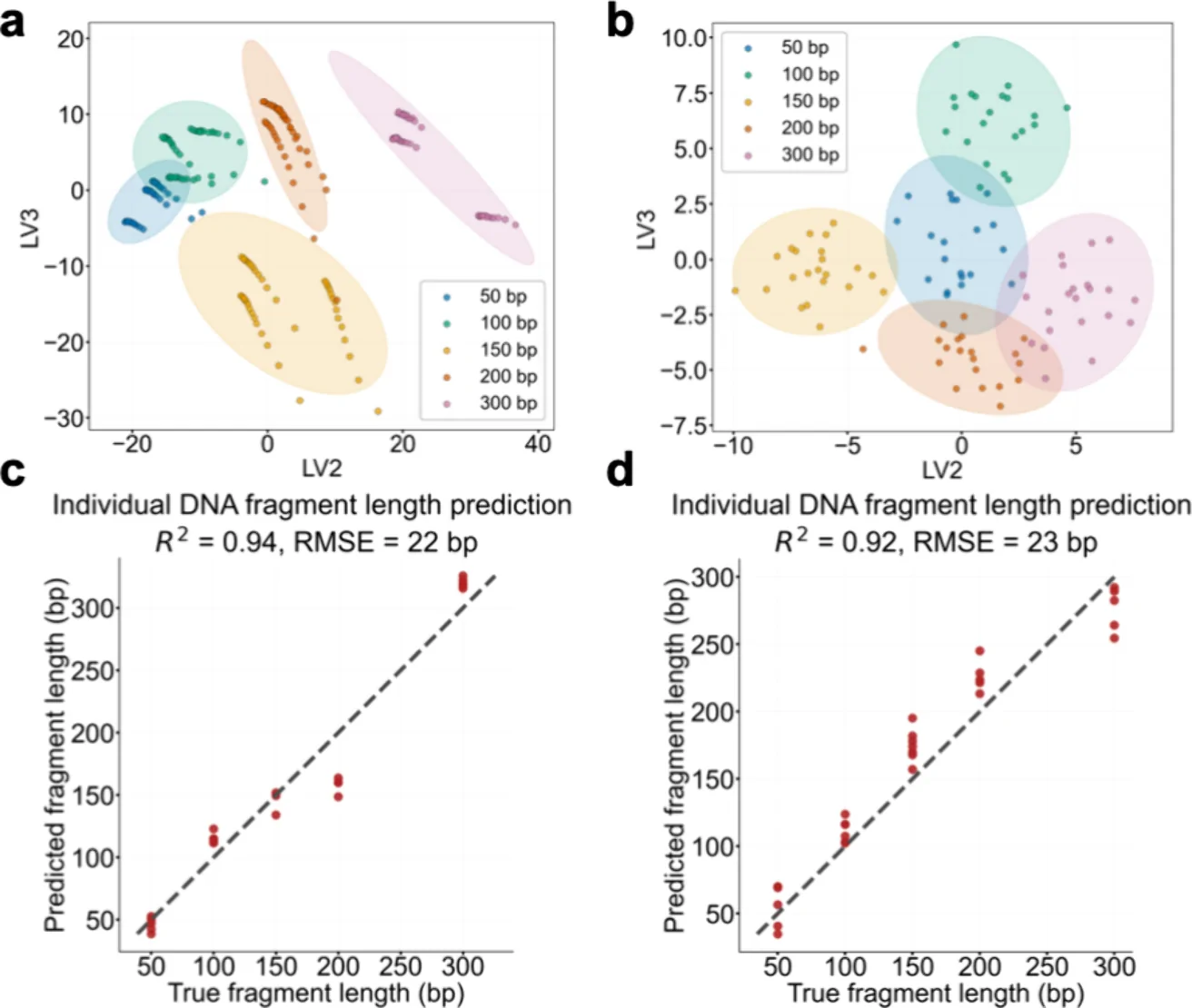
Spectroscopic and machine learning analysis of individual DNA fragment length. Partial Least Squares (PLS) scores plot of (a) FTIR and (b) Raman data. Solutions with different fragment lengths cluster based on their spectral features. Scatter plots showing the performance of PLSR models trained on (c) FTIR and (d) Raman data. Ellipses in (a) and (b) represent the 95% confidence interval for each group.

Following this analysis, we developed Partial Least Squares Regression (PLSR) models to predict DNA fragment length directly from the spectra. To ensure that the models focused on the most relevant biochemical information, the analysis was restricted to the strongest DNA-specific vibrational windows (750–1800 cm^–1^ for ATR-FTIR and 600–1800 cm^–1^ for Raman). The ATR-FTIR data set contained 135 spectra in total, comprising five fragment lengths, three independent replicate preparations per fragment length, and nine spectral measurements per replicate. The Raman data set contained 150 spectra in total, comprising five fragment lengths, two physical laser spot positions per fragment length, and 15 spectra collected from each position. Data splitting in the PLSR models followed this experimental structure to prevent data leakage: for ATR-FTIR, the first two replicate preparations were used for training and cross-validation, while the third replicate was retained as the independent test set; for Raman, the split was performed by physical laser spot position, as described in the Methods. Models were optimized by selecting the number of latent variables based on cross-validation performance, with Van der Voet’s F-test applied to prevent overfitting (, and Methods).

Both PLSR models trained independently using ATR-FTIR and Raman spectra achieved comparable performance on their respective test sets. For ATR-FTIR, the model achieved an R^2^ of 0.94 with an RMSE of 22 bp, with a 95% bootstrap confidence interval ranging from 15.05 to 28.42 bp (Figure 3c). The Raman model yielded an *R* ^2^ of 0.92 and an RMSE of 23 bp, with a 95% bootstrap confidence interval ranging from 18.50 to 28.05 bp (Figure 3d).

To identify the spectral regions contributing most strongly to fragment-length prediction, regression coefficients from the ATR-FTIR and Raman PLSR models were plotted as a function of wavenumber (). In the ATR-FTIR model, the largest negative coefficients occurred within 1700–1730 cm^–1^, associated mainly with carbonyl vibrations, as well as around 1450 cm^–1^ within the nucleobase associated region. The strongest positive coefficients were concentrated around 1180–1250 cm^–1^, encompassing the phosphate-backbone, while additional coefficient features occurred within 800–950 cm^–1^, where sugar phosphate-associated vibrations are observed. The Raman coefficient profile contained a greater number of features across the spectral range. The largest coefficient magnitudes occurred around 1500–1650 cm^–1^, associated mainly with nucleobase vibrations; 1100–1200 cm^–1^ and 900–1050 cm^–1^, containing phosphate-backbone contributions; and 650–850 cm^–1^, where base ring-breathing modes are commonly observed, with additional contributions throughout the 1250–1450 cm^–1^ nucleobase- associated region.

In summary, these results show that statistical modeling of ATR-FTIR and Raman spectra enables accurate length quantification of monodisperse DNA solutions containing a single length. While this demonstrates that fragment-length-associated spectral variation can be captured under controlled conditions, the present analysis did not explicitly assess the effect of DNA sequence composition. Further validation using fragments of matched length but differing GC/AT content will therefore be required to determine the robustness of the approach to sequence-dependent spectral variation.

### Data Fusion Enhances Fragment Length Prediction

We next integrated ATR-FTIR and Raman spectra using data fusion (Methods). Although both techniques detect molecular vibrations, ATR-FTIR measures dipole moment changes while Raman spectroscopy measures polarizability changes. As a result, when the techniques are used together they have been shown to be complementary improving predictive performance.^29–31^ For example, while the observed symmetric PO_2_^–^ stretch (∼1080 cm^–1^) was a dominant, length sensitive feature in FTIR, this was weaker in the Raman spectrum. Conversely, the O–P–O associated backbone vibration (∼785 cm^–1^) was a dominant peak in Raman but was almost absent in FTIR.

To fully evaluate whether integrating data from both modalities improved model fit, we first created a fused data set using low level feature concatenation. For each sample, the Raman spectrum (originally 600–1800 cm^–1^) was appended to the end of the FTIR spectrum (originally 750–1800 cm^–1^) to create a single, unified data vector (Figure 4a). Applying the PLSR methodology developed for the independent modalities to this fused data set improved the model’s predictive ability. By combining spectral data from ATR-FTIR and Raman spectroscopies, the data fusion model achieved an R^2^ of 0.96 and reduced the RMSE to 17 bp (Figure 4b), outperforming the FTIR only model (RMSE 22 bp) and the Raman only model (RMSE 23 bp).

**Figure 4:**
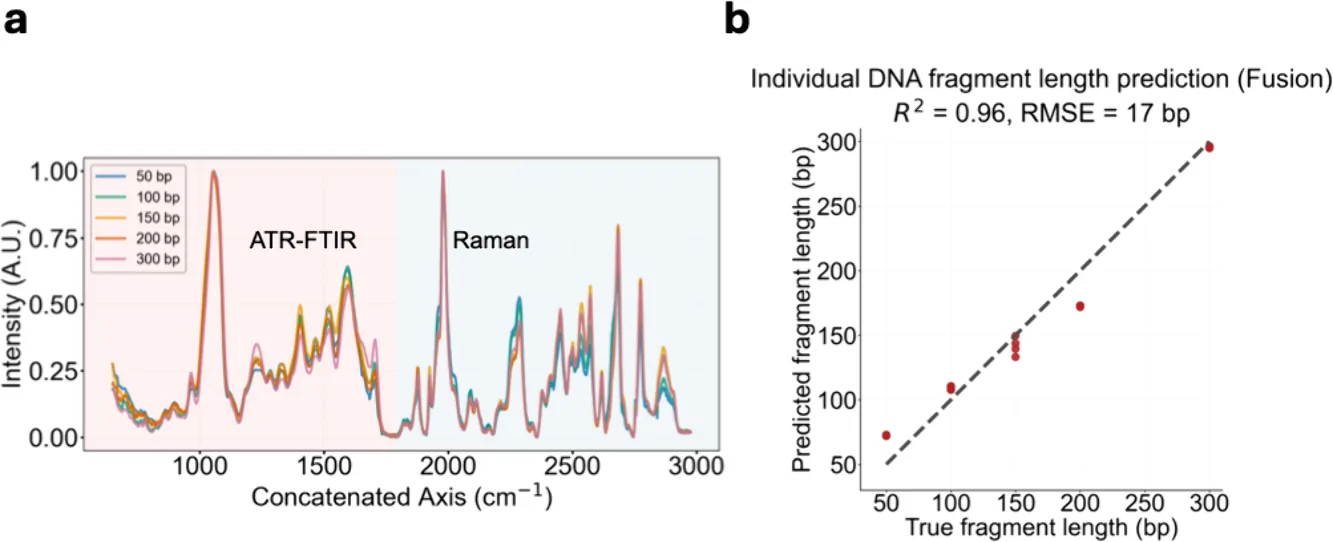
Data fusion of FTIR and Raman spectra increases predictive accuracy. (a) Visualization of low level feature concatenation. The *x* axis represents a synthetic axis created for continuity, showing the FTIR spectrum (up to ∼ 1800 cm^–1^) and the Raman spectrum (from ∼1800 cm^–1^ onward). (b) Regression plot for a PLSR model trained on the fused data set.

This work represents the first application of multimodal vibrational spectroscopy for quantitative DNA fragment length analysis, introducing a methodology that combines complementary ATR-FTIR and Raman data to improve prediction accuracy in monodisperse DNA solutions.

### Developing a 1D-CNN Model on Polydisperse Mixtures with Discrete DNA Fragment Lengths

We next sought to determine whether fragment-length information could be deconvoluted from polydisperse DNA solutions containing discrete DNA fragment sizes. We generated 29 polydisperse mixtures comprising combinations of up to five fragment lengths: 50, 100, 150, 200, and 300 bp, in systematically varying proportions. These were analyzed alongside five monodisperse samples, one for each fragment length, giving a complete sample set of 34 compositions that included monodisperse, binary, ternary, and quinary mixtures (Figure 5a, , and Methods). For each composition, ATR-FTIR spectra were collected from three independent replicates, with nine scans acquired per replicate, resulting in a total of 918 spectra. This provided a comprehensive data set for training and validating the fragment-length model. These polydisperse mixtures produce complex, overlapping spectral features that are not readily resolved using linear models alone ().

**Figure 5:**
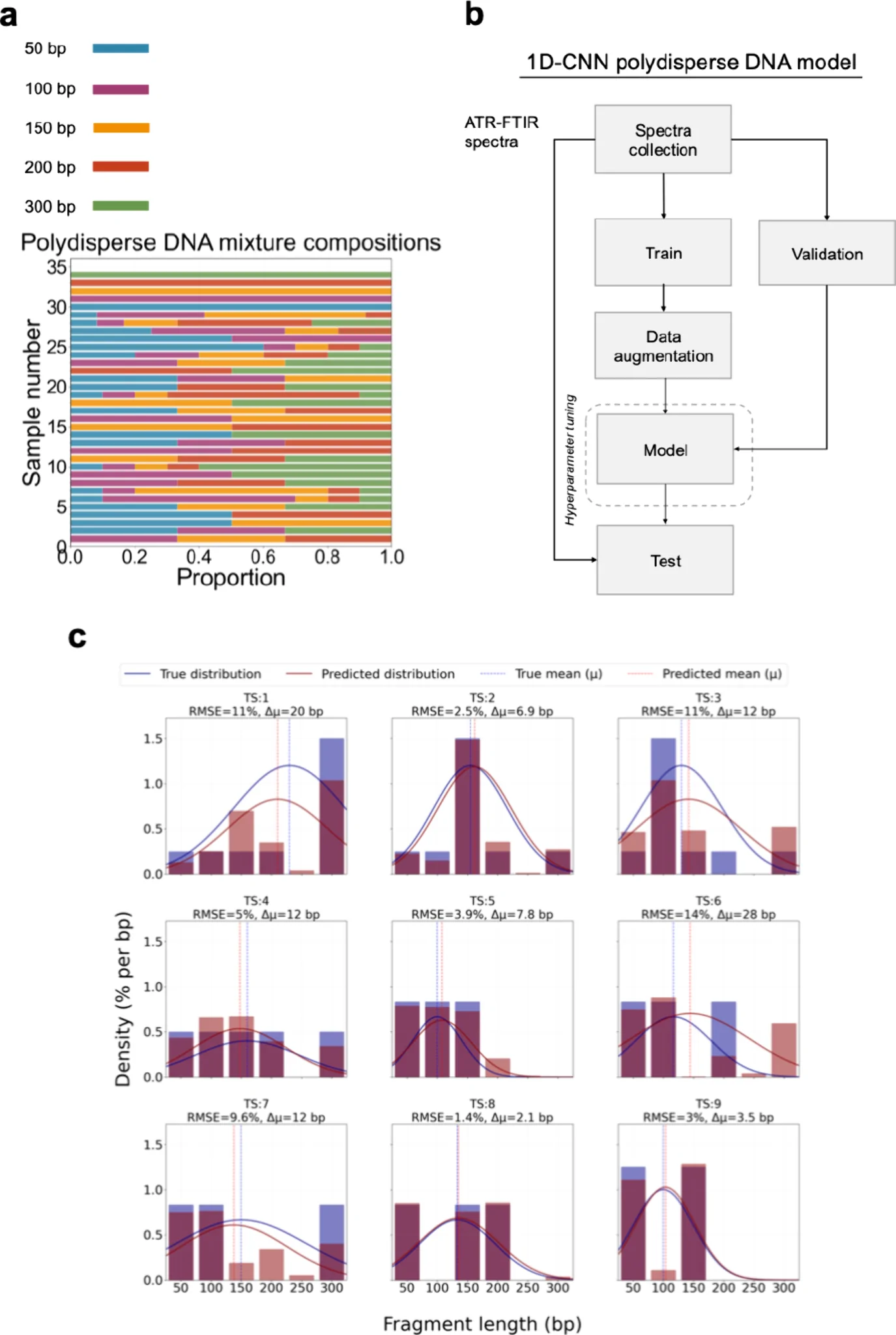
1D-CNN workflow and performance on polydisperse DNA mixtures containing discrete fragment lengths. (a) Stacked bar chart showing the composition of 34 DNA sample compositions generated by mixing DNA solutions containing fragments of defined lengths (50 bp, 100 bp, 150 bp, 200 bp, and 300 bp). Each bar represents a unique mixture composition, with colors indicating the proportion of each fragment length present. (b) Machine learning workflow for model development. (c) Predicted fragment length distributions produced by the 1D-CNN model (red bars), with the corresponding Gaussian estimate overlaid as a solid red line. True fragment length probabilities, derived from the known mixture compositions, are shown as blue bars with their corresponding Gaussian estimates indicated by solid blue lines. Results are shown for nine representative samples from the independent test set (TS). Vertical dotted lines indicate the weighted mean fragment length for both predicted (red) and true (blue) distributions.

To address this, we developed a 1D-CNN (Figure 5b). This deep learning architecture is optimized for identifying local patterns in spectral data and capable of disentangling spectral overlap.^32–35^ The 1D-CNN was trained on the measured ATR-FTIR spectra from the DNA mixtures for simultaneous prediction of all fragment percentages. Following a similar train and test data partitioning method as the PLSR models, the first two replicate spectra measurements were used for training, with 20% of the training reserved for validation, while the third replicate set served as an independent test.

To improve generalization and reduce overfitting, the training set was augmented 6-fold using intensity scaling, baseline shifts, and the addition of low amplitude noise (). Model optimization was performed by minimizing the loss () between the predicted and true fragment proportions on the training set, with validation loss used to monitor convergence and generalization.

As the model outputs discrete proportions for each fragment class, we further represented the predicted mixtures as continuous Gaussian distributions using their weighted mean and standard deviation. These Gaussian representations were used for visualization and comparison, and not as a constraint during training. This smoothing step provided a more intuitive basis for comparing fragmentation patterns between samples, particularly when assessing mixtures enriched in shorter versus longer fragments where fragmentation profiles can serve as diagnostic biomarkers.^4,36,37^

The 1D-CNN achieved good accuracy in DNA length proportion prediction across the test set (). The lowest error was for 50 bp fragments (RMSE = 4.7%) and the highest for 300 bp fragments (RMSE = 9.0%), where RMSE was computed across all individual test sample spectra for each fragment class independently. Across all fragment classes, the model achieved a mean per-class RMSE of 7.3%, calculated as the average of the five per-class RMSEs (50, 100, 150, 200, and 300 bp). The average shift between the true and predicted weighted distribution means (Δμ) was 11 bp, calculated per mixture composition in the test set, then averaged across all test mixtures. To further assess model generalization to mixture compositions not included during training, the trained model was evaluated on three unseen DNA mixtures. The predicted fragment-length distributions closely followed the true distributions, with per-mixture RMSE values of 3.0–3.8% ().

In summary, these results suggest that a 1D-CNN trained on augmented spectral data can identify spectral features associated with mixtures of DNA containing different fragment lengths and provide approximate reconstruction of the underlying length distributions. These findings provide an initial proof-of-concept for applying machine learning-assisted vibrational spectroscopy to DNA fragmentomic profiling under controlled mixture conditions.

### Quantifying Complex DNA Mixtures with Continuous Fragment Lengths

We next explored whether our methodology could be extended to estimate fragment length distributions in biological DNA samples comprising continuous, rather than discrete, fragment lengths. To do this, we prepared 11 sheared DNA samples spanning a range of fragment length distributions and quantified their fragment length distribution using gel electrophoresis, the current gold standard (Methods). Gel electrophoresis confirmed that all samples contained broad and continuous fragment length distributions rather than discrete populations. Mean fragment lengths ranged from ∼195 to ∼240 bp, while median lengths spanned from ∼175 bp to ∼235 bp. Quantitative binning of the electropherograms revealed overlap across neighboring fragment lengths for all samples, with the most occurring fragment typically centered within the 150–200 bp and 200–250 bp bins (). In general, shorter fragments (<100 bp) were consistently present at low abundance while longer fragments (250–350 bp) increased progressively across the sample series.

We subsequently acquired ATR-FTIR spectra from the DNA samples, where for each biological DNA sample, spectra were collected from three independent replicates, with nine scans acquired per replicate, resulting in a total of 297 spectra. Given the limited number of biological samples, the data set was divided at the sample level into six samples for model training and five independent samples for testing, with both subsets harboring comparable DNA fragment length distributions. We employed a transfer learning strategy rather than training a new model from scratch (Figure 6a). Specifically, the 1D-CNN model previously trained on the 34 purified compositions was repurposed as a feature extractor, and its final layers were fine-tuned using the biological training set. This two-stage approach enabled the model to adapt to the domain shift between purified and biological DNA spectra while retaining the spectral features learned from the larger original data set.

**Figure 6:**
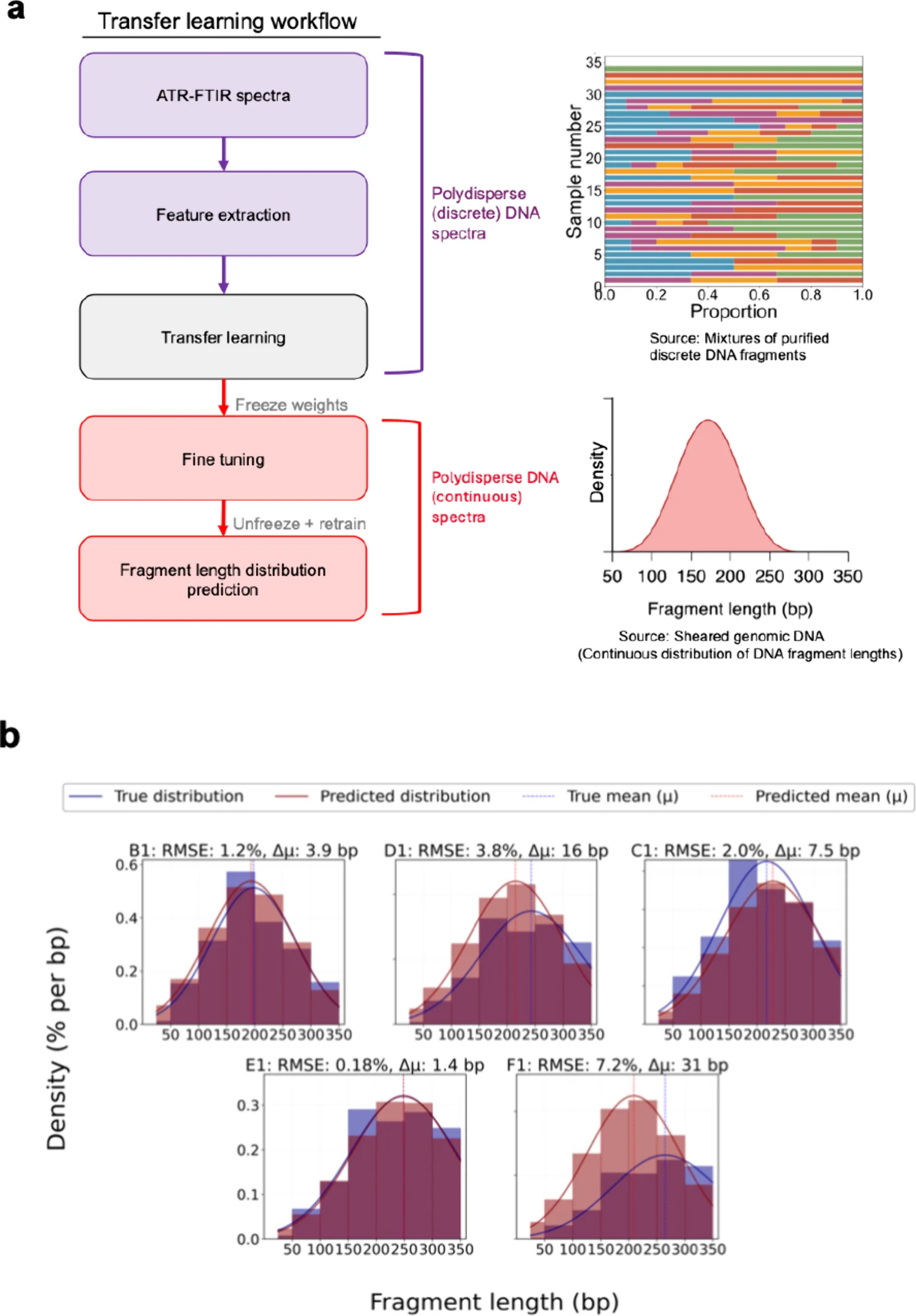
Predicting DNA fragment length in complex DNA polydisperse samples. (a) Transfer learning workflow for extending fragment length prediction from discrete polydisperse DNA mixtures to biological DNA samples containing continuous fragment length distributions. A 1D-CNN was pretrained on pure DNA ATR-FTIR spectra with augmented spectra acquired from mixtures of purified DNA fragments with discrete lengths combined in defined proportions. The pretrained network was then repurposed as a feature extractor, and model weights were fine-tuned using a small set of biological samples containing sheared genomic DNA, enabling adaptation from the purified domain (discrete fragment predictions) to the biological domain (continuous fragment length distributions) and prediction of fragment length distributions in heterogeneous DNA samples. (b) Testing on the independent test samples. The transfer learning approach yields good alignment between the predicted (red) and expected (blue) base pair distributions.

The transfer learning model was trained to perform multioutput regression, predicting the proportion of DNA across seven fragment length bins (25–50 bp, 50–100 bp, 100–150 bp, up to 300–350 bp) using the reference data obtained via gel electrophoresis for these same samples. The fine-tuned model was then applied to the averaged ATR-FTIR spectra from the five unseen biological samples (B1-F1) in the test set. The compositions of these samples are detailed in . As shown in Figure 6b, the transfer learning framework was able to recover the overall fragment-length distributions for most of the held-out biological samples. Performance was evaluated by computing the RMSE between the predicted and reference percentage values across the seven fragment-length bins for each test sample, followed by averaging the per-sample RMSE values across the five test samples. This gave a mean per-sample RMSE of 2.9%. The highest per-sample RMSE was observed for sample F1, with an RMSE of 7.2% and a 95% bootstrap confidence interval ranging from 3.4% to 10.3%. The lowest per-sample RMSE was observed for sample E1, with an RMSE of 0.18% and a 95% bootstrap confidence interval ranging from 0.15% to 0.21%. In addition, the average absolute shift in distribution center, Δμ, between the predicted and gel electrophoresis-derived reference distributions was approximately 12 bp across the five test samples.

In summary, these results show that transfer learning enabled spectral models trained on purified DNA and derived augmented spectra to estimate fragment length profiles in biological samples containing polydisperse DNA. The ability to recover fragment length distributions in sheared biological DNA samples extends the approach beyond controlled purified mixtures and provides an initial demonstration that the spectral features learned from defined DNA standards can be transferred to more complex sample types.

## Conclusions

In this study, we provide an initial proof of principle that vibrational spectroscopy combined with machine learning can be used to estimate DNA fragment length distributions without prior separation or labeling. Using ATR-FTIR and Raman spectroscopy, length-dependent spectral variation was observed in monodisperse DNA samples spanning 50–300 bp. PLSR regression coefficient analysis indicated that model predictions were influenced by multiple spectral regions, including phosphate-backbone-associated bands, nucleobase vibrations, and regions linked to DNA conformational or hydrogen-bonding changes. In both ATR-FTIR and Raman models, several of the strongest positive coefficient features occurred within phosphate- and backbone-associated regions, suggesting that these vibrations likely contributed to fragment-length prediction. One possible explanation is that shorter DNA fragments have a higher density of terminal ends per unit mass, which may increase the relative contribution of terminal and near-terminal backbone environments. These structural differences could influence phosphate-associated vibrational bands while being less directly dependent on base composition than nucleobase-associated regions. Additionally, multimodal data fusion of ATR-FTIR and Raman improved upon either modality alone, quantifying individual fragment lengths with an RMSE of 17 bp.

Building on these findings, we extended the approach to the simultaneous quantification of multiple fragment lengths in polydisperse mixtures using ATR-FTIR exclusively, where length-dependent spectral changes were more clearly resolved. A 1D-CNN trained on augmented spectra from 34 purified DNA mixtures successfully learned fragment-specific spectral representations, and transfer learning to a small cohort of biological samples with continuous fragment length distributions enabled accurate prediction on five independent test samples, with an average RMSE of 2.9%. Future work should therefore expand the training data by including more fragment lengths to define the practical resolution limit of the approach, while also incorporating greater sequence diversity to determine how base composition influences model performance. Nevertheless, this work provides proof of principle that vibrational spectroscopy can serve as a rapid, label-free, nondestructive platform for fragment length analysis, with potential to support fragmentomic profiling in genomic workflows and circulating tumor DNA diagnostics.

## Supplementary Material













## Data Availability

The data for this work are accessible through the University of Southampton Institutional Research Repository via 10.5258/SOTON/D3838 (2026).

## Notes

Supporting Figures 1–12 with legends describing PLSR regression coefficients, latent variable optimization, data augmentation strategies, 1D-CNN model training, prediction performance, and transfer learning loss curves; legends for Supporting Tables 1–5; Supporting References (PDF)
Table S1. ATR-FTIR peak assignments and supporting references for spectral bands identified in the ATR-FTIR spectra, covering wavenumber ranges and corresponding molecular vibration assignments (XLSX)
Table S2. Raman peak assignments and supporting references for spectral bands identified in the Raman spectra, covering wavenumber ranges and corresponding molecular vibration assignments (XLSX)
Table S3. Composition of purified DNA fragment mixtures used for model development; volumetric ratios (μL) of 50, 100, 150, 200, and 300 bp fragments across 34 single, binary, ternary, quaternary, and five-component mixtures prepared using an extreme vertices experimental design (XLSX)
Table S4. Continuous fragment length distribution profiles (25–350 bp bins, % contribution) for biological DNA samples in the training cohort, derived from gel electrophoresis electropherograms, with calculated weighted mean and median fragment lengths (XLSX)
Table S5. Quantitative fragment length distribution profiles (25–350 bp bins, % contribution) for biological DNA samples in the independent test cohort (B1–F1), derived from gel electrophoresis electropherograms, with calculated weighted mean and median fragment lengths (XLSX)
